# Contrast-enhanced ultrasonography for early prediction of response of neoadjuvant chemotherapy in breast cancer

**DOI:** 10.3389/fonc.2022.1026647

**Published:** 2022-12-01

**Authors:** Jiabao Guo, Bao-Hua Wang, Mengna He, Peifen Fu, Minya Yao, Tian’an Jiang

**Affiliations:** ^1^ Department of Ultrasound Medicine, The First Affiliated Hospital, School of Medicine, Zhejiang University, Hangzhou, Zhejiang, China; ^2^ Department of Breast Surgery, The First Affiliated Hospital, School of Medicine, Zhejiang University, Hangzhou, Zhejiang, China

**Keywords:** ultrasonography, neoadjuvant chemotherapy, breast cancer, response, vascular heterogeneity

## Abstract

Neoadjuvant chemotherapy (NAC) is widely accepted as a primary treatment for inoperable or locally advanced breast cancer before definitive surgery. However, not all advanced breast cancers are sensitive to NAC. Contrast-enhanced ultrasonography (CEUS) has been considered to assess tumor response to NAC as it can effectively reflect the condition of blood perfusion and lesion size. Therefore, this study aimed to evaluate the diagnostic performance of CEUS to predict early response in different regions of interest in breast tumors under NAC treatment. This prospective study included 82 patients with advanced breast cancer. Parameters of TIC (time-intensive curve) between baseline and after the first cycle of NAC were calculated for the rate of relative change (Δ), including Δpeak, ΔTTP (time to peak), ΔRBV (regional blood volume), ΔRBF (regional blood flow) and ΔMTT (mean transit time). The responders and non-responders were distinguished by the Miller-Payne Grading (MPG) system and parameters from different regions of tumors were compared in these two groups. For ROI 1(the greatest enhancement area in the central region of the tumor), there were significant differences in Δpeak1, ΔRBV1 and ΔRBF1 between responders and non-responders. For ROI 2 (the greatest enhancement area on edge of the tumor), there were significant differences in Δpeak2 and ΔRBF2 between the groups. The Δpeak1 and ΔRBF2 showed good prediction (AUC 0.798-0.820, p ≤ 0.02) after the first cycle of NAC. When the cut-off value was 0.115, the ΔRBF2 had the highest diagnostic accuracy and the maximum NPV. Quantitative TIC parameters could be effectively used to evaluate early response to NAC in advanced breast cancer.

## Introduction

Neoadjuvant chemotherapy (NAC) is widely accepted as the primary treatment for inoperable or locally advanced breast cancer before definitive surgery. The benefits of NAC in terms of overall survival and improvement in quality of life have been verified in many clinical trials and studies and have been able seen turning inoperable tumors into operable tumors and providing the option of breast-conserving surgery instead of mastectomy (1). However, not all locally advanced breast cancers are sensitive to NAC. Studies have indicated that almost 10-35% of patients were insensitive to chemotherapy drugs, meaning that these patients experienced disease progression during the period of NAC (2–4). Therefore, the ability to predict early response to initial cycles and replace drugs with alternative agents in non-responders would be of considerable clinical significance (5).

Magnetic resonance imaging (MRI) is the best method for assessing the tumor response to NAC, for its performance was generally superior to that of mammography, ultrasonography (US), and clinical examination in a meta-analysis with 300 patients (6). Nevertheless, some studies suggest that MRI gadolinium-based contrast agents diffuse from the blood vessels into adjacent interstitial tissues, overestimating the extent of the residual tumor (7–10).

Changes in blood vessels in breast lesions are known to occur before morphological changes (11), and contrast-enhanced ultrasonography (CEUS) can effectively reflect the condition of blood perfusion and lesion size due to its ability to obtain macrovascular and microvascular information about the lesions (12, 13). CEUS is a quantitative kinetic imaging modality that offers the time-intensity curve (TIC) before and after NAC treatment to aid our understanding of the complexity of angiogenesis in breast tumors (14, 15).

Previous studies on breast cancer have explored changes in tumor size, that usually occur after the second cycle of NAC, so earlier predictors reflecting angiogenesis and metabolic activity may change before tumor shrinkage (16, 17). The viability of CEUS to predict tumor response after completing the first cycle of NAC is unknown (11). Several studies have suggested that CEUS could predict early response to NAC (18–22). However, the heterogeneity of tumor vessels has been ignored and the different regions of interest inside the tumor need to be discussed. The difference between the CEUS features of the marginal zone and central region in breast cancer deserves attention. Therefore, it was crucial to highlight the characteristics of CEUS in the marginal zone and central region of breast cancer in this study.

In this study, we performed a comparative analysis of the relative variation ratio of quantitative TIC parameters from different regions of tumors between responders and non-responders to investigate the potential role of CEUS in evaluating the early response to NAC in breast cancer patients.

## Material and methods

### Clinical materials

This prospective study was approved by the ethics committee of the First Affiliated Hospital of Zhejiang University (Hangzhou, China). All patients provided written informed consent. A total of 82 female patients diagnosed with stage II or III unilateral breast cancer and scheduled to receive NAC were recruited for this study at the First Affiliated Hospital of Zhejiang University (Hangzhou, China) between May 2019 and May 2022.

### Chemotherapy regimen

Prior to surgery, there were two main NAC regimens for all patients in this study: (1) anthracycline-based regimens and (2) taxane regimens. Then the duration of NAC was mainly 6 or 8 cycles. In addition, HER2-positive patients were treated with trastuzumab. The treatment protocol and timeline followed the guidelines provided by NCCN and China Anti-Cancer Association (CACA). Drug treatment for 21 days was considered 1 cycle and an interval of 20 days occurred following before the initiation of the next round of chemotherapy. Image examinations were performed before the second NAC cycle. Surgical excision was performed within 20 days after 6 or 8 cycles of drug treatment.

### CEUS examination

All patients underwent the CEUS before NAC, after the first cycle of NAC. An ESAOTE MyLab ClassC ultrasound diagnostic instrument (Esaote SpA, Genoa, Italy) was performed for CEUS. The ultrasound contrast agent SonoVue (59 µg; Bracco SpA, Milan, Italy) was added to 5 ml saline, and a milky microbubble suspension was generated by vigorous agitation. The breast was first scanned with B-mode and CDFI to identify the tumor location and detect its vascularity. Choosing the largest section of the tumor, a real-time contrast-enhanced US imaging using a low mechanical index ranging between 0.06 and 0.08 was performed. A total of 4.8 ml SonoVue suspension was rapidly injected through an anterior elbow vein and then 5 ml of saline was injected to flush the tube. When the transducer was stabilized with minimal pressure, images were recorded with a clip function for 120 secs. Contrast observation continued until the lesion-enhanced image disappeared.

### Image review and data analysis

**Figure 1:**
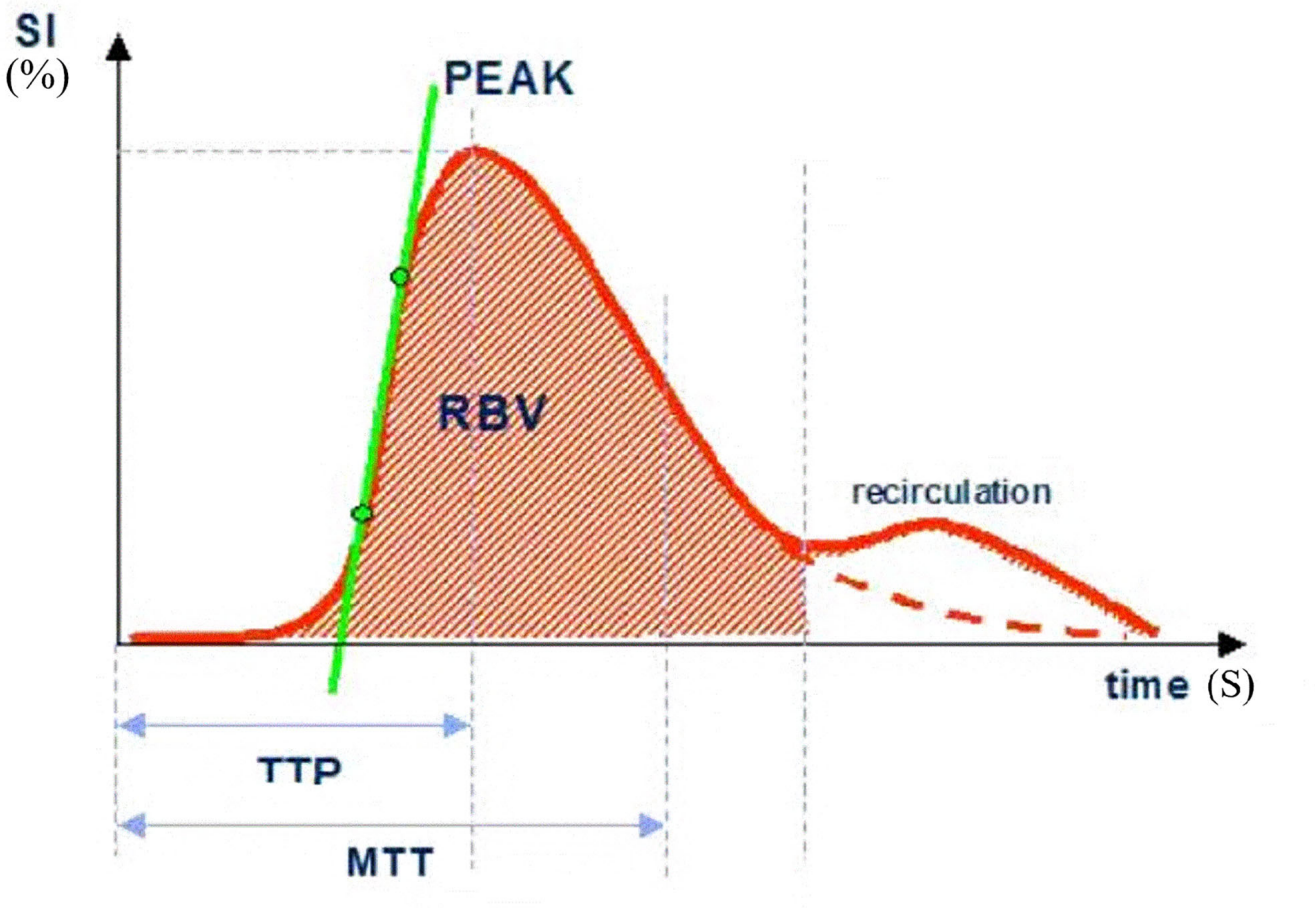
SI, signal intensity; TTP, time to peak; MTT, mean transit time; Peak, peak intensity (%); RBV, regional blood volume; RBF, regional blood flow (RBV/MTT).

The postprocessing analysis of the data was performed quantitatively by two senior physicians, with more than 5 years and 10 years of experience in breast imaging. The gold standard-postoperative pathological diagnosis was assessed by the Miller-Payne Grading (MPG) system (described in further detail below). TIC was generated from the region of interest (ROI), in which quantitative blood perfusion parameters, including peak percent (peak), time to peak (TTP), regional blood volume (RBV), regional blood flow (RBF) and mean transit time (MTT) were compared in responders and non-responders. The detailed explanations of above TIC parameters were described in **Figure 1**. The different regions of breast cancer are defined as follows: central region: the region with a diameter of 0.5 cm in the center of the lesion. If the lesion is small, the sampling frame can be appropriately reduced; marginal zone: the boundary of the enhanced range of lesions was taken as the external area. The relative variation ratio (Δ) in the parameters after the first cycle of NAC vs. baseline were calculated as follows: Δ= (parameter_pre_-parameter_1st_)/parameter_pre_.

### Pathological evaluation

The pathology was assessed by the Miller-Payne Grading (MPG) system, which compares the cancer cellularity of the core needle biopsy (before NAC) with the resected tumor (23–25). 1): no reduction in overall cellularity, 2): a minor loss of tumor cells (up to 30% loss), 3): 30-90% loss of malignant cells, 4): more than 90% loss of malignant cells, and 5): no identifiable malignant cells, although ductal carcinoma may be presented *in situ*. 1)-3) were defined as “non-response”, while 4)-5) were defined as “response”.

### Statistical analysis

The data were analyzed by SPSS version 17.0 statistics software (IBM Corp., Armonk, NY, USA). The measurement data was checked by Student’s t-test, which was expressed as $\bar{x}$ ±s. The count data were evaluated by the Chi-square test. To investigate inter-observer agreement and intra-observer reliability, we evaluated both Pearson correlation coefficients and Cronbach’s Alpha. Receiver operating characteristic (ROC) curves and the area under the curve (AUC) were obtained to evaluate the performance of perfusion parameters to predict early response after NAC. The range of 0.9–1.0 indicates an excellent predictor; 0.8–0.9, a good predictor; 0.7–0.8, a general predictor; and< 0.7, a poor predictor (25). The optimal threshold (cut-off) was chosen according to the Youden index. A p value<0.05 was considered to indicate a statistically significant difference.

## Results

### Clinical characteristics of the patients

**Figure 2:**
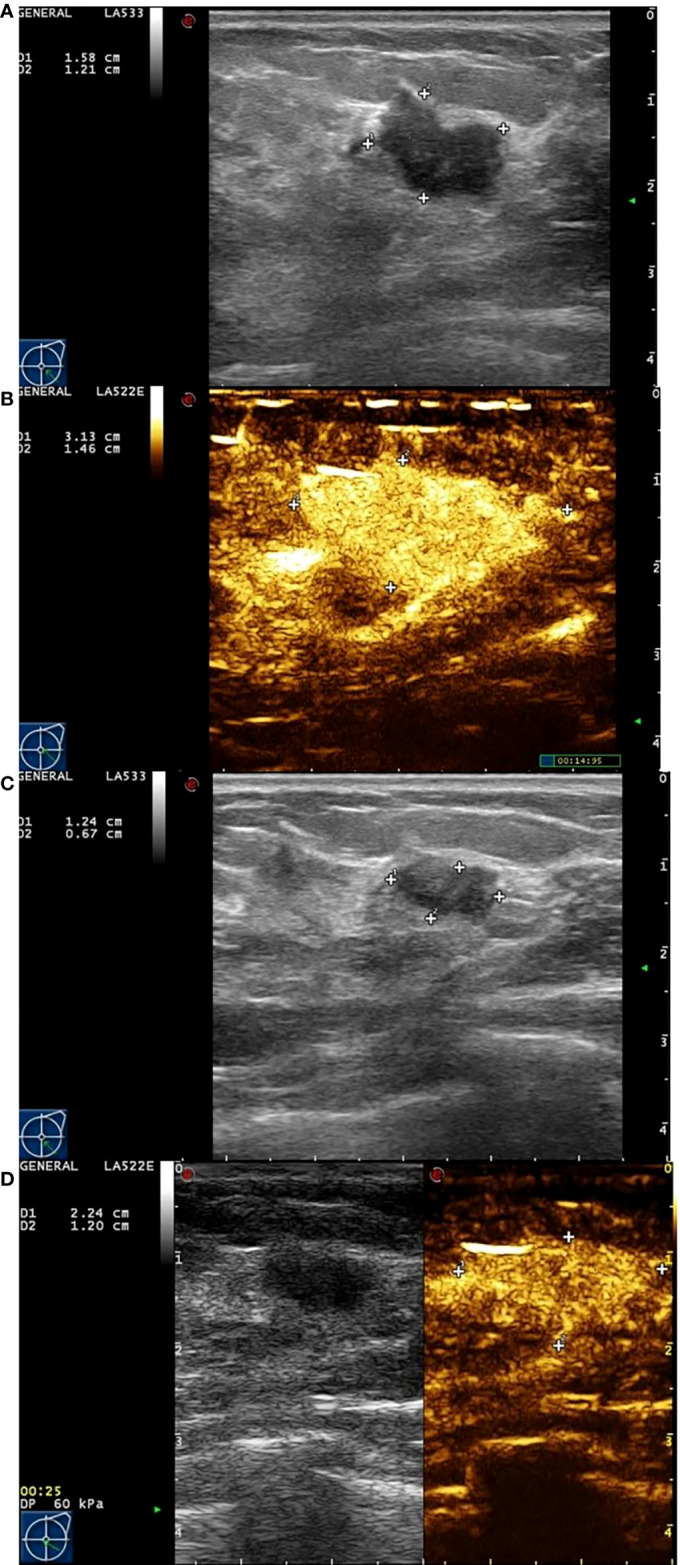
Images from a patient of non-response to NAC. **(A)** At baseline before NAC, the tumor size was measured by ultrasonography. **(B)** At baseline before NAC, the tumor size was measured by contrast-enhanced ultrasound. The extent of tumor was significantly larger than that of ultrasonography. **(C)** After the last cycle of NAC before surgery, the tumor size was measured by ultrasonography. **(D)** After the last cycle of NAC before surgery, the tumor size was measured by contrast-enhanced ultrasound. Although the extent of tumor has shrunken, there still have large number of contrast agents inside.

**Figure 3:**
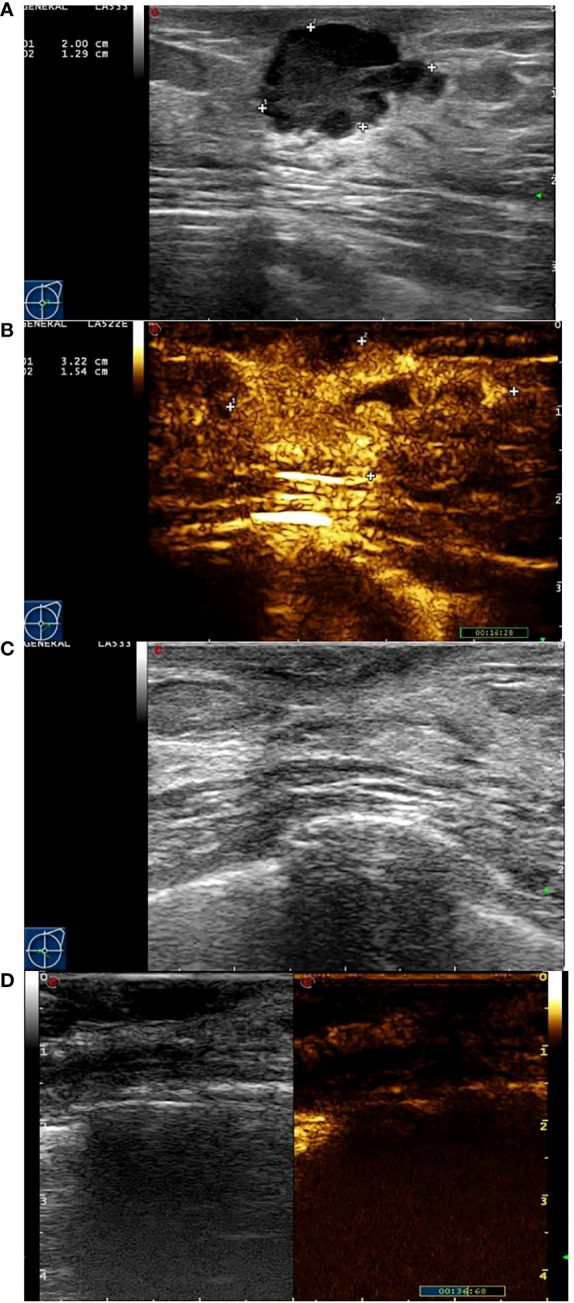
Images from a patient of response to NAC. **(A)** At baseline before NAC, the tumor size was measured by ultrasonography. **(B)** At baseline before NAC, the tumor size was measured by contrast-enhanced ultrasound. The extent of tumor was significantly larger than that of ultrasonography. **(C)** After the last cycle of NAC before surgery, the tumor has disappeared fundamentally by ultrasonography. **(D)** After the last cycle of NAC before surgery, there was not area of high enhancement fundamentally.

Eighty-two female patients with a mean age 47.5±10.8 (30 to78) years who received NAC and surgery were recruited for this present study. The mean tumor diameter measured by ultrasound was 2.78±1.42cm (1.03 cm to 5.63 cm). All patients, including 45 cases (45 lesions) of infiltrating ductal carcinoma, 28 cases (28 lesions) of infiltrating lobular carcinoma and 9 case (9 lesion) of mucinous carcinoma were confirmed by postoperative pathology. 54 of the 82 patients showed a response (Miller-Payne score 4 or 5) and 28 showed non-response (Miller-Payne score 1, 2, or 3) (**Figures 2**, **3**). There were no significant differences between the clinical characteristic parameters of these two groups (**Table 1**).

**Table 1:** Clinicopathological features of the patients at baseline.

characteristic	Response (54)	Non-response (28)	P-value
Age (years)	47.0±10.14	49.1±11.47	0.169
Tumor maximum diameter (cm)	3.15±1.21	2.96±1.53	0.456
histology			0.470
MC	7 (13.0%)	2 (7.1%)	
IDC	29 (53.7%)	16 (57.1%)	
ILC	18 (33.3%)	10 (35.7%)	
Tumor subtype			0.205
Luminal A	21 (30.6%)	11 (39.3%)	
Luminal B	17 (25%)	9 (32.1%)	
HER-2 positive	12 (38.9%)	5 (17.9%)	
TNBC	4 (5.6%)	3 (10.7%)	

MC, mucinous carcinoma; IDC, infiltrating ductal carcinoma; ILC, infiltrating lobular carcinoma; TNBC, triple negative breast carcinoma.

### Agreement and reliability of perfusion parameters

The results were compared by independent analysis of two senior physicians. All perfusion parameters had high inter-observer and intra-observer repeatability (r>0.886, p<0.001, Cronbach’s Alpha>0.936).

### Comparison of the relative variation ratio of quantitative TIC parameters

**Figure 4:**
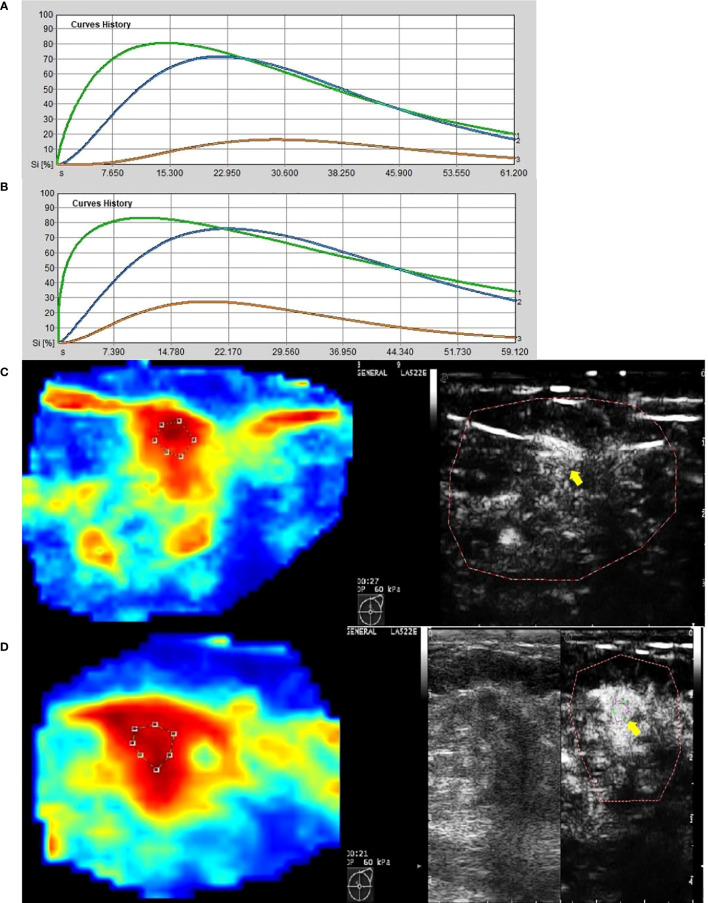
Images from a patient of non-response to NAC. **(A)** At baseline before NAC, contrast-enhanced ultrasound imaging showed a time-intensity curve generated according to regions of interest. The green line represents ROI 1 (the greatest enhancement area in central region of tumor), the blue line represents ROI 2 (the greatest enhancement area in edge of tumor), the yellow line represents normal breast tissue. **(B)** After the first cycle of NAC, neither ROI 1 nor ROI 2 declined significantly. **(C)** At baseline before NAC, the circle represents ROI 1, and the red area means rich blood supply; the right image was gray-scale which correspond to the left one, and the yellow arrow referred to ROI 1. **(D)** After the first cycle of NAC, the circle represents ROI 1, and the red area expanded, instead of shrunk; the right image was gray-scale which correspond to the left one, and the yellow arrow referred to ROI 1.

**Figure 5:**
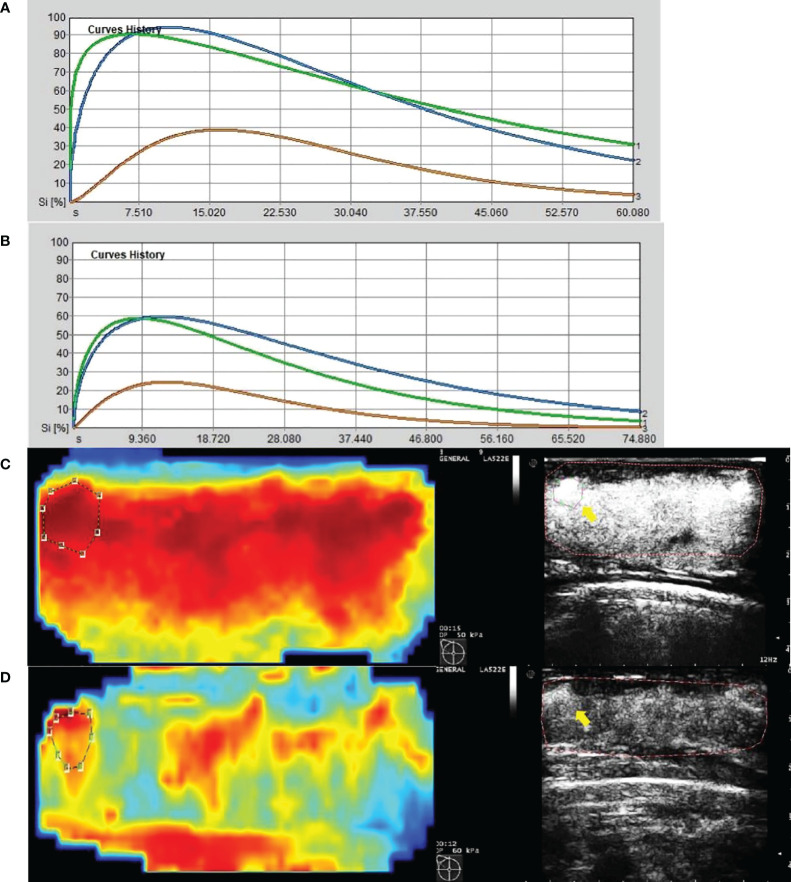
Images from a patient of response to NAC. **(A)** At baseline before NAC, contrast-enhanced ultrasound imaging showed a time-intensity curve generated according to regions of interest. The green line represents ROI 1(the greatest enhancement area in central region of tumor), the blue line represents ROI 2(the greatest enhancement area in edge of tumor), the yellow line represents normal breast tissue. **(B)** After the first cycle of NAC, both ROI 1 and ROI 2 declined significantly. **(C)** At baseline before NAC, the circle represents ROI 2, and the red area means rich blood supply; the right image was gray-scale which correspond to the left one, and the yellow arrow referred to ROI 2. **(D)** After the first cycle of NAC, the circle represents ROI 2, and the red area shrunk significantly; the right image was gray-scale which correspond to the left one, and the yellow arrow referred to ROI 2.

The data in **Table 2** were obtained by analysis of ROI 1, which represents the greatest enhancement area in the central region of the tumor, and ROI 2, which indicates the greatest enhancement area in the edge of the tumor. The data show us that there were significant differences in Δpeak1, ΔRBV1, and ΔRBF1 after the first cycle of NAC between responders and non-responders (p-values were 0.001, 0.012, and 0.002 respectively). No statistically significant difference was found for ΔTTP 1 and ΔMTT1 (p-values were 0.068, and 0.056 respectively). It was observed that Δpeak2 and ΔRBF2 after the first cycle of NAC in responders were higher than those of non-responders (p-values were 0.000 and 0.003 respectively). Other parameters, including ΔTTP2, ΔRBV2, and ΔMTT2, had no significant difference (**Figures 4**, **5**).

**Table 2:** TIC parameters in ROI 1 and ROI 2 after 1st Cycle of NAC for Discrimination between Responders and Non-responders.

		Response	Non-response	P-value
	n	54	28	
ROI 1	Δpeak1	0.17±0.13	0.01±0.17	0.001
	ΔTTP1	-0.40±0.67	-0.01±0.52	0.068
	ΔRBV1	0.31±0.31	-0.07±0.60	0.012
	ΔRBF1	0.18±0.13	0.03±0.14	0.002
	ΔMTT1	0.19±0.31	0.10±0.50	0.056
ROI 2	Δpeak2	0.17±0.14	-0.01±0.12	0.000
	ΔTTP2	-0.26±0.53	-0.05±0.45	0.218
	ΔRBV2	0.10±0.52	-0.15±0.55	0.169
	ΔRBF2	0.16±0.18	-0.02±0.13	0.003
	ΔMTT2	0.01±0.51	-0.12±0.47	0.446

Δ, the relative variation ratio; ROI 1, the greatest enhancement area in central region of tumor; ROI 2, the greatest enhancement area in edge of tumor.

### Early predictors of tumor NAC response

**Table 3** present the diagnostic performance of each statistically significant predictor for early response of NAC. After the first cycle of NAC, Δpeak1 and ΔRBF2 showed good prediction (AUC 0.806-0.820, p ≤ 0.02). ΔRBF1, ΔRBV1 and Δpeak2 showed general prediction (AUC 0.725-0.798, p ≤ 0.032). The sensitivity, specificity, PPV, NPV and accuracy of the cut-off value of each statistically significant predictor for early response of NAC were analyzed in **Table 4**. ΔRBF2 with a cut-off value of 0.115 had the highest diagnostic accuracy and the maximum NPV.

**Table 3:** Diagnostic Performance of TIC parameters in ROI 1 and ROI 2 to Predict Response after 1st cycle of NAC.

variable	Cut-off	AUC	SE	95%CI	P-value
Δpeak1	>0.075	0.806	0.071	(0.668, 0.945)	0.020
ΔRBV1	>0.412	0.725	0.081	(0.567, 0.884)	0.020
ΔRBF1	>0.065	0.798	0.076	(0.649, 0.947)	0.002
Δpeak2	>0.137	0.769	0.080	(0.612, 0.926)	0.005
ΔRBF2	>0.115	0.820	0.068	(0.687, 0.953)	0.001

Cut-off, the optimal threshold; AUC, area under ROC curve; SE, standard error; CI, confidence interval; ROC, receiver operating characteristic.

**Table 4:** Comparison of sensitivity, specificity, accuracy, PPV and NPV (%) of cut-off value of parameters between ROI 1 and ROI 2.

Cut-off	ROI 1			ROI 2	
	Δpeak1>0.075	ΔRBV1>0.412	ΔRBF1>0.065	Δpeak2>0.137	ΔRBF2>0.115
sensitivity	84.6%	61.5%	69.2%	61.5%	88.5%
specificity	78.6%	92.9%	92.9%	100%	78.6%
PPV	88%	94.1%	94.7%	100%	88.5%
NPV	73.3%	56.5%	61.9%	58.3%	78.6%
accuracy	82.5%	72.5%	77.5%	75%	85%

Cut-off, the optimal threshold; ROI 1, the greatest enhancement area in central region of tumor; ROI 2, the greatest enhancement area in edge of tumor.

## Discussion

Neoadjuvant chemotherapy has been recognized as a crucial method to decrease tumor cells and significantly increase the rate of breast-conserving and surgical resection (26). However, not every patient who underwent the NAC gets treatment benefits, because the effective rate of NAC ranges from 60-90%. Hence, assessing early response to treatment is key to ensure success in NAC.

A previous study revealed that there was an imbalance in the spatial distribution of tumor blood vessels (27). The microvascular density around the tumor was higher than that in the center, and the necrotic and cystic area was lower than the central areas. This is tumor vascular heterogeneity. CEUS features of breast cancer have regional distribution differences, which are due to the heterogeneity of the tumor. As the front of tumor invasion, the marginal zone of breast cancer has special biological characteristics and may be more sensitive to drugs than the central region. In this study, the perfusion parameters of the central region and the marginal zone were studied separately.

The multiple parameters, Δpeak1, ΔRBV1, and ΔRBF1, after the first cycle of NAC in ROI 1, were larger in responders than non-responders (p<0.05). The peak was an enhancement description index for blood perfusion assessment. That means when the tumor has more macrovascular inside, more contrast agents stay in the vessels, leading to a high peak value. Our study proved that the value of Δpeak1 increased significantly after the first cycle of NAC, especially in responders, which is consistent with the results of Amioka et al. (18). RBV is a quantitative parameter representing regional blood volume, which can reflect the blood supply inside the lesion. Before NAC, the vascularity of malignant lesions was rich, twisted, and easy to form arterio-venous fistula, which would present a higher enhancement in tumors. Effective NAC can shrink vessels providing nutrients to the tumor and reduce the number of new blood vessels. That might explain why ΔRBV1 increased significantly after the first cycle of NAC in responders. RBF was an index indicating regional blood flow, which was calculated by RBV/MTT, closely related to the patency of blood flow inside the tumor. Chemotherapy-induced changes such as necrosis, sclerosis, or inflammation can obstruct contrast agents’ flow in the original tumor site (28), which would lead to an increase in the ΔRBF1 value.

Δpeak2 and ΔRBF2, obtained from ROI 2 after the first cycle, were significantly higher in responders than non-responders (p<0.05). Some studies (11, 12, 29) have explained that different regions within the same malignant lesion can have different characteristics because of tumor heterogeneity. We know that large and rich nourishing vessels in the tumors provide nutrients, whereas tumor angiogenesis as well as new tumor tissue formation are often at the edge of the lesion to infiltrate surrounding normal tissues. Unlike ROI 1, there are abundant new expansive microvascular with wall thin, lack of muscular layer, direction circuity, and formation of arteriovenous fistula in ROI 2, which lead to high concentration contrast agents in tumor vascular bed. With the marginal vein lymphatic tumor emboli in formation, however, interstitial edema became more serious, leading to slower perfusion of contrast agents relative to the central region and resulting in turbulence in blood flow (30–32). That might explain why ΔRBF2 increased after the first cycle of NAC in responders. These parameters from different ROI highlighted that the optimal ROI positioning would have brought more accurate predictors. The microbubble agents in the CEUS only stayed within blood vessels, and the new vessels at the edge were richer than those in the center. The loss of basement membrane resulted in increased vascular permeability, and formed abundant anastomosis, which led to the contrast agent turbulence in blood flow (30–32). Hence, ΔRBF2 was the most accurate indicator.

TTP is the time from zero intensity to the peak, and MTT is the mean transit time. Some researchers (20) observed longer TTP in responders compared to non-responders after two cycles of NAC. In our research, however, there was no significant difference in TTP and MTT between responders and non-responders. The reason may be that TTP and MTT will be changed effectively until after two cycles of NAC.

To our knowledge, this is the first study to assess the value of TIC at different ranges including the edge and central regions of the lesion for early prediction of the efficiency of NAC in breast cancer using CEUS. In this study, we observed some meaningful changes after the first cycle of NAC. Δpeak1 and ΔRBF2 were potential criteria to predict the early response of NAC on breast cancer. This study could be of great clinical significance and further in-depth research on different regions of lesions with TIC could help predict the early response in breast cancer tumors under NAC.

Our study had several limitations. First, the study population was too small. A large-scale study with a standardized method is still needed. Second, the histopathology and tumor subtype of these breast cancer recruited were heterogeneous. Third, even though the TIC parameters calculated by contrast software had excellent inter-observer and intra-observer repeatability and reliability, the CEUS examination was performed only once before NAC and after the first cycle of NAC for each patient.

In conclusion, quantitative TIC parameters can be effectively used to evaluate early response to NAC in advanced breast cancer.

## Data availability statement

The original contributions presented in the study are included in the article/supplementary material. Further inquiries can be directed to the corresponding author.

## Ethics statement

Written informed consent was obtained from the individual(s) for the publication of any potentially identifiable images or data included in this article.

## Author contributions

TJ designed the experiments. MH, PF, and MY collected the data. JG and B-HW conducted the experiments and analyzed the data. All authors contributed to the article and approved the submitted version.

## Funding

This study was supported by the Foundation of Zhejiang Natural Science Committee, grant No LQ20H180013.

## Conflict of interest

The authors declare that the research was conducted in the absence of any commercial or financial relationships that could be construed as a potential conflict of interest.

## Publisher’s note

All claims expressed in this article are solely those of the authors and do not necessarily represent those of their affiliated organizations, or those of the publisher, the editors and the reviewers. Any product that may be evaluated in this article, or claim that may be made by its manufacturer, is not guaranteed or endorsed by the publisher.
